# Surgical Presentations of Eosinophilic Gastroenteritis: A Case Report

**DOI:** 10.7759/cureus.80144

**Published:** 2025-03-06

**Authors:** Taher Faydi, Maha Shomaf, Mais Kadri, Ahmad Kadri, Kareem Kadri

**Affiliations:** 1 Vascular Surgery, Northampton General Hospital NHS Trust, Northampton, GBR; 2 Pathology, Jordan University Hospital, Amman, JOR; 3 Diabetes and Endocrinology, East Lancashire Hospitals NHS Trust, Leyland, GBR; 4 Gastroenterology, Nottingham University Hospitals NHS Trust, Nottingham, GBR; 5 Internal Medicine, University Hospitals of North Midlands, Stoke-on-Trent, GBR

**Keywords:** acute abdominal symptoms, eosinophilic gastroenteritis, eosinophilic infiltration, rare gastrointestinal disorders, surgical presentation

## Abstract

Eosinophilic gastroenteritis (EGE) is a rare disorder characterized by eosinophilic infiltration of the gastrointestinal (GI) tract without any definitive cause of eosinophilia. It presents with various non-specific GI symptoms, depending on the affected site and layer of involvement, often leading to delayed diagnosis and treatment. While the primary treatment consists of oral corticosteroids, extreme cases may necessitate surgical intervention.

We present a case of a 50-year-old female who arrived at the emergency department with a three-day history of vomiting, diffuse abdominal pain, and tachycardia. She underwent emergency surgery due to acute abdominal symptoms. Intraoperatively, a mass-like lesion was identified in the distal antrum of the stomach, causing pyloric narrowing. Histopathological examination confirmed EGE. The patient had previously undergone laparoscopic cholecystectomy and open appendectomy for abdominal pain, which we now believe were misdiagnosed as separate surgical pathologies when they were possibly early manifestations of EGE.

After an extensive literature review, this may be the first reported case of EGE in Jordan and the first case requiring a surgical procedure in the country.

## Introduction

First described in 1937 by Kaijser, interest in eosinophilic gastroenteritis (EGE) has grown in recent years in parallel with an increasing number of case reports from different continents [1]. The prevalence is poorly described in the literature. One study conducted in the United States estimated the standardized prevalence at approximately 6.3 per 100,000 for eosinophilic gastritis, 8.4 per 100,000 for EGE, and 3.3 per 100,000 for eosinophilic colitis [2]. However, this may represent only the tip of the iceberg, as EGE is likely underdiagnosed due to the significant variation in clinical manifestations and the fact that patients often do not seek medical attention for mild or intermittent symptoms. The pathogenesis and etiology of EGE remain unclear [3]. An allergic disorder is present in approximately half of the patients [4], yet extensive studies have failed to identify a reproducible allergic reaction to specific foods in all cases. Thus, the role of allergy as a stimulus for eosinophil recruitment to the gastrointestinal tract remains controversial [4].

No standardized diagnostic criteria for EGE exist, but certain findings support the diagnosis. Talley et al. [5] identified three main diagnostic criteria: (1) the presence of gastrointestinal symptoms; (2) biopsies demonstrating eosinophilic infiltration; and (3) no evidence of parasitic or extraintestinal disease. Peripheral eosinophilia has been reported as uniformly associated with EGE [3].

The diagnosis of EGE can be made in most cases based on clinical suspicion in the appropriate context, along with endoscopic or full-thickness biopsy or paracentesis [6]. It is preferable to obtain at least six biopsy specimens from both normal and abnormal areas of the bowel. Gross features of this disease include prominent mucosal folds, hyperemia, ulceration, or nodularity, while histopathology typically demonstrates increased numbers of eosinophils (often >50 eosinophils per high-power field) [7].

The currently accepted classification for EGE was proposed by Klein et al. [8] in 1970. It includes three subtypes: (1) mucosal type: the most prevalent subtype, characterized by non-specific abdominal symptoms such as abdominal pain, nausea, vomiting, diarrhea, fecal occult blood, anemia, and weight loss. Some studies suggest a potential bias in its reported prevalence due to the relative ease of obtaining endoscopic biopsies from the mucosal layer [9]. (2) Muscularis type: often presents with gastrointestinal obstructive symptoms mimicking pyloric stenosis or gastric outlet syndrome. (3) Subserosal type: frequently associated with increased abdominal volume (e.g., due to ascites) and eosinophilia. This subtype generally responds well to corticosteroid therapy [10].

Treatment with corticosteroids remains the cornerstone of EGE management. While unnecessary surgery should be avoided, surgical intervention may sometimes be required when a definitive diagnosis cannot be established or when complications such as obstruction or perforation occur [11]. This was the case for our patient, in whom urgent surgical treatment was necessary due to an acute surgical abdomen.

## Case presentation

A 50-year-old female patient presented to the emergency department complaining of diffuse abdominal pain that began five days prior to presentation. The pain initially started gradually, was intermittent, and mild in severity. However, four days later, it became severe (preventing the patient from sleeping), constant, and was associated with multiple episodes of projectile vomiting. Vomiting occurred 20-30 minutes after meals, and the pain mildly improved after each episode. She did not report any changes in bowel habits or other gastrointestinal symptoms. However, she mentioned experiencing similar but milder abdominal pain for the last 10 years, for which she had never sought medical attention. Instead, she self-medicated with over-the-counter proton pump inhibitors (PPIs) and oral analgesics.

Her past medical history included diabetes for the past eight years and hypertension for the last four years, both controlled with oral medication. Her past surgical history included an uncomplicated laparoscopic cholecystectomy 13 years ago for multiple gallbladder stones and an appendectomy six years ago for abdominal pain. The pathological examination of the appendix at that time was unremarkable for any inflammation.

Her vital signs at presentation are shown in Table 1. Her head, neck, and chest examinations were unremarkable. An abdominal examination revealed healthy-looking scars from previous surgeries but was notable for guarding and rigidity on palpation. Bowel sounds were normal on auscultation, and her digital rectal examination was unremarkable. Her initial laboratory results are shown in Table 2.

**Table 1:** Vital parameters at presentation.

Parameter	Obtained value	Reference range
Heart rate	109 bpm	60–100 bpm
Respiratory rate	29 breaths/min	12–20 breaths/min
Blood pressure	105/65 mmHg	100/60–120/80 mmHg
Temperature	37.1°C	36.1–37.2°C
Oxygen saturation	96% on room air	≥95% on room air

**Table 2:** Laboratory investigation results.

Test	Obtained value	Reference range
Hemoglobin (Hb)	11 g/dL	12–16 g/dL (female)
White blood cell count (WBC)	13.9 × 10⁹/L	4–11 × 10⁹/L
Neutrophils	65%	40–70%
Lymphocytes	25%	20–45%
Monocytes	7%	2–10%
Eosinophils	3%	1–6%
Platelet count	215 × 10⁹/L	150–450 × 10⁹/L
Creatinine	0.74 mg/dL	0.6–1.2 mg/dL
Amylase	91 U/L	30–110 U/L

An abdominal ultrasound revealed gross thickening of the pyloric canal wall, forming a mass-like lesion measuring 5 cm in diameter, associated with a moderate amount of free fluid in the pelvic cavity. A small quantity of fluid was also observed in Morrison’s pouch, along with generalized thickening of the small bowel wall. An abdominal X-ray was unremarkable, showing no signs of air-fluid levels or free air under the diaphragm.

The patient demonstrated symptoms of an acute surgical abdomen with intra-abdominal fluid collection. The initial impression was a perforated duodenal or gastric ulcer. After primary resuscitation and obtaining informed consent, she was taken to the operating room for an emergency laparotomy. A midline incision was made, and intraoperative exploration revealed approximately 400 mL of serous fluid in the abdomen. A mass-like lesion was identified in the distal antrum of the stomach, causing the narrowing of the pyloric region. No perforation or other pathology was observed. A resection of the antrum, including the mass-like lesion, was performed, followed by a gastrojejunostomy and a jejunojejunostomy drainage procedure (Omega procedure).

**Figure 1:**
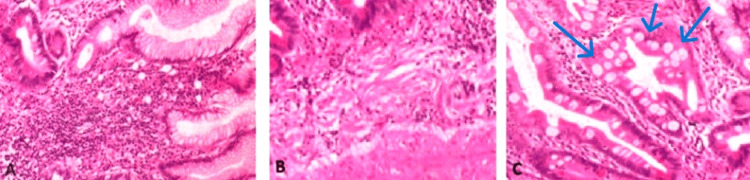
Microscopic images of the specimen. (A) The mucosal layer and (B) the muscularis mucosae showing an increased number of eosinophils in hematoxylin and eosin stain (HE, 400×). (C) Microscopic image showing the presence of goblet cells and intestinal metaplasia in the mucosal layer.

Histopathological examination of the surgical specimen revealed a thickening of the stomach wall with pyloric stenosis on gross inspection. Microscopic examination showed a significant increase in eosinophils (>50 per high-power field) in both the mucosal layer (Figure 1A) and the muscularis mucosae (Figure 1B), along with intestinal metaplasia and the presence of goblet cells (Figure 1C).

**Figure 2:**
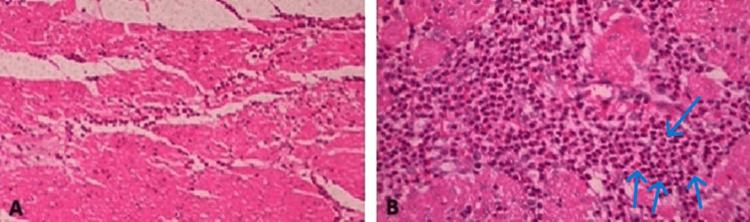
Microscopic images of the muscularis layer with eosinophilic infiltration (>50 per high-power field). (A) At 400× magnification and (B) at 1000× magnification.

Additionally, eosinophilic infiltration with a significant number of eosinophils (>50 per high-power field) was noted in the muscularis layer (Figure 2), which was associated with hypertrophy and was determined to be the cause of the patient's pyloric stenosis and symptoms.

The patient had an uneventful postoperative recovery and was discharged five days later. Stool analysis was negative for ova and parasites. She was started on corticosteroid therapy and remains symptom-free.

## Discussion

EGE is not only a rare disease but also one with a wide range of clinical symptoms and presentations, often mimicking other conditions, making an accurate diagnosis challenging for physicians. The most common symptoms include abdominal pain, nausea, vomiting, and diarrhea. The surgical presentation as gastric outlet obstruction has been documented in medical literature. However, because this presentation is extremely rare, it is often not considered in the differential diagnosis. This is a crucial factor, as early and accurate diagnosis of this disease may allow for effective treatment, potentially avoiding the need for surgery [10].

This case describes a patient who presented to the emergency department with an acute surgical abdomen. The initial differential diagnosis included a perforated viscus, most likely in the antrum of the stomach, with a duodenal ulcer perforation also considered. Malignancy and its complications could not be ruled out, given the presence of an antral mass-like lesion causing obstruction and the accumulation of serous fluid in the abdomen. Gastric malignancies account for approximately 35% of all gastric outlet obstruction cases [12].

EGE is typically diagnosed via upper GI endoscopy with biopsy, which confirms the presence of eosinophilic infiltration in the pathology examination. Proper diagnosis of EGE can prevent unnecessary surgery, as the condition responds well to corticosteroid therapy. However, in this case, the urgency of the presentation prevented further preoperative investigations, and the diagnosis was only confirmed after surgical resection. The patient was subsequently started on corticosteroid therapy. We suspect that her previous symptoms and surgeries (cholecystectomy and appendectomy) may have been manifestations of EGE affecting her gastrointestinal tract, mimicking appendicitis and biliary colic. However, no definitive proof exists to confirm this theory, and only limited evidence is available in the literature on this topic.

Few cases of EGE presenting as pyloric stenosis have been reported in children [13,14]. Additionally, five case reports describe gastric outlet obstruction due to EGE in adults; four patients were successfully treated with corticosteroids, showing good clinical response [15-18], while one patient underwent subtotal gastrectomy with gastrointestinal reconstruction using the Roux-en-Y technique [10].

## Conclusions

EGE is often misdiagnosed, leading to unnecessary surgical interventions. In our case, urgent surgical intervention was required due to acute symptoms, and the diagnosis was only confirmed postoperatively through histopathological examination. This postoperative confirmation was crucial because it indicated the necessity of initiating corticosteroid therapy, as recurrence can occur even after surgical resection.

Due to our limited understanding of EGE's natural history, long-term follow-up is essential for monitoring disease progression and treatment response. Further research is needed to ensure early recognition and optimal treatment of this rare condition.

## References

[1] Méndez-Sánchez N, Chávez-Tapia NC, Vazquez-Elizondo G, Uribe M (2007). Eosinophilic gastroenteritis: a review. Dig Dis Sci.

[2] Jensen ET, Martin CF, Kappelman MD, Dellon ES (2016). Prevalence of eosinophilic gastritis, gastroenteritis, and colitis: estimates from a national administrative database. J Pediatr Gastroenterol Nutr.

[3] Caldwell JH (2002). Eosinophilic gastroenteritis. Curr Treat Options Gastroenterol.

[4] Harfoushi K (2008). Eosinophilic gastrointestinal disorders. Jordan Med J.

[5] Talley NJ, Shorter RG, Phillips SF, Zinsmeister AR (1990). Eosinophilic gastroenteritis: a clinicopathological study of patients with disease of the mucosa, muscle layer, and subserosal tissues. Gut.

[6] Haberkern CM, Christie DL, Haas JE (1978). Eosinophilic gastroenteritis presenting as ileocolitis. Gastroenterology.

[7] Breiteneder H, Mills EN (2005). Molecular properties of food allergens. J Allergy Clin Immunol.

[8] Klein NC, Hargrove RL, Sleisenger MH, Jeffries GH (1970). Eosinophilic gastroenteritis. Medicine (Baltimore).

[9] Oh HE, Chetty R (2008). Eosinophilic gastroenteritis: a review. J Gastroenterol.

[10] Leal R, Fayad L, Vieira D (2014). Unusual presentations of eosinophilic gastroenteritis: two case reports. Turk J Gastroenterol.

[11] Yun MY, Cho YU, Park IS, Choi SK, Kim SJ, Shin SH, Kim KR (2007). Eosinophilic gastroenteritis presenting as small bowel obstruction: a case report and review of the literature. World J Gastroenterol.

[12] Chowdhury A, Dhali GK, Banerjee PK (1996). Etiology of gastric outlet obstruction. Am J Gastroenterol.

[13] Snyder JD, Rosenblum N, Wershil B, Goldman H, Winter HS (1987). Pyloric stenosis and eosinophilic gastroenteritis in infants. J Pediatr Gastroenterol Nutr.

[14] Choi SJ, Jang YJ, Choe BH, Cho SH, Ryeom H, Hong SJ, Lee D (2014). Eosinophilic gastritis with gastric outlet obstruction mimicking infantile hypertrophic pyloric stenosis. J Pediatr Gastroenterol Nutr.

[15] Lombardi C, Salmi A, Passalacqua G (2011). An adult case of eosinophilic pyloric stenosis maintained on remission with oral budesonide. Eur Ann Allergy Clin Immunol.

[16] Bachmeyer C, Ammouri W, Ravet N (2006). Pyloric stenosis in an adult with eosinophilic gastroenteritis. (Article in French). Rev Med Interne.

[17] Chou CH, Shin JS, Wu MH, Chow NH, Lin XZ (1996). Eosinophilic gastroenteritis with esophageal involvement. J Formos Med Assoc.

[18] Tursi A, Rella G, Inchingolo CD, Maiorano M (2007). Gastric outlet obstruction due to gastroduodenal eosinophilic gastroenteritis. Endoscopy.

